# Preparation and application evaluation of monoclonal antibodies against Monkeypox virus A29 protein

**DOI:** 10.3389/fmicb.2025.1547021

**Published:** 2025-01-31

**Authors:** Ai Xiangjun, Zhang Xinlan, Xu Ye, Tan Chufan, Duan Chen, Liao Nami, Liu Junxi, Qiu Yilan, Hou Defu, Wang Qinglin, Liu Rushi

**Affiliations:** ^1^College of Medical Technology and Translational Medicine, Hunan Normal University, Changsha, China; ^2^Hunan Xuxiang Biotechnology Co., Ltd., Changsha, China; ^3^School of Life Sciences, Hunan Normal University, Changsha, China

**Keywords:** Monkeypox virus (MPXV), monoclonal antibodies, chemiluminescence enzyme immunoassay, colloidal gold immunochromatography, antigen detection

## Abstract

Monkeypox virus (MPXV) is a DNA virus belonging to the Orthopoxvirus genus of the Poxviridae family. It causes symptoms similar to Smallpox virus and is a zoonotic virus with widespread prevalence. Antigen detection is a fast and effective detection method. The MPXV A29 protein not only plays an important role in the virus lifecycle but also serves as a promising target for developing specific antibodies, which have significant potential for application in the diagnosis of MPXV. The coding sequences of the MPXV A29 protein, Cowpox virus (CPXV) 163 protein homolog and Vaccinia virus (VACV) A27 protein homolog were chemically synthesized, and all three recombinant proteins were expressed in *Escherichia coli* (BL21 Star). Then, the recombinant A29 protein was used as an antigen to immunize BALB/c mice, and a total of 4 monoclonal antibodies against A29 protein were obtained. Using two homologous proteins as reverse screening systems, a specific monoclonal antibody, mAb-25, against the A29 protein was screened. Then, the mAb-25 was used as a coating antibody to pair with other monoclonal antibodies, leading to the identification of a well-matched antibody pair. A chemiluminescence enzyme immunoassay (CLEIA) and immunochromatographic gold assay were subsequently established using the optimal antibody pair. The experimental results indicate that monoclonal antibodies against the A29 protein hold significant potential for application in the diagnosis of MPXV.

## 1 Introduction

MPXV was first identified in 1958, when the Danish Serum Institute discovered vesicular pustule lesions resembling smallpox in captive imported Javanese macaques (Magnus et al., 1959; Giradkar and Khatib, 2022). The pathogen was subsequently isolated from these lesions, marking the first recorded detection of MPXV (Rahmani et al., 2024). In 2022, monkeypox cases were reported in 70 countries worldwide, prompting World Health Organization (WHO) to declare the outbreak a public health emergency of international concern (Singh et al., 2024). In August 2024, WHO declared the monkeypox outbreak the second global public health emergency in two years, underscoring the significant threat of MPXV to human health.^1^ MPXV infection can result in a range of clinical symptoms, including fever, headache, muscle pain, lymphadenopathy, and a distinctive rash that progresses from papules to pustules and ultimately to scabs, causing considerable physical and mental distress to affected individuals (Adegboye et al., 2021). Most patients are able to recover on their own within a period of time, but for individuals with weakened immune function, the symptoms may be more severe and even lead to death, with a mortality rate of up to 10% (Adegboye et al., 2024).

From the perspective of the structural characteristics, MPXV is a DNA virus belonging to Orthopoxvirus genus of the Poxviridae family (Karagoz et al., 2023). Mature MPXV particles are composed of multiple structurally distinct components, including an outer membrane, surface tubules, two lateral bodies, a large double-stranded linear DNA genome (approximately 197 kb in length), and a double concave dumbbell-shaped nucleoprotein (Huang et al., 2022). The pathogenic process of MPXV infection is usually divided into three stages: incubation period, prodromal period, and rash period (Gessain et al., 2022). The entire course of the illness, from the onset of symptoms to the shedding of scabs, may be contagious (Halani et al., 2022). MPXV has multiple modes of transmission, all of which are closely related to direct contact with infected animals or humans, including transmission through respiratory droplets, placental transmission, direct contact with skin abrasions or pollutants (Karagoz et al., 2023). Currently, the availability of vaccines for monkeypox remains limited. In September 2024, after the MPXV spread from the Democratic Republic of Congo to its neighboring countries, WHO has declared the MVA-BN vaccine as the first monkeypox vaccine to be included on the prequalification list. However, the MVA-BN vaccine is currently not approved for use in individuals under the age of 18.^2^ Due to the easy transmission and widespread prevalence of MPXV, early diagnosis and timely intervention of MPXV are particularly important to control the spread of the epidemic effectively.

The laboratory detection methods for monkeypox include electron microscopy observation, cell culture, nucleic acid detection, antigen detection, and antibody detection (Resman Rus et al., 2024). Antigen detection is a highly accurate and effective method that can yield results within minutes to hours, making it especially valuable in clinical applications. MPXV is a large DNA virus with a genome encoding approximately 200 proteins, which makes the development of antigen immunoassay relatively complex due to the diversity of protein species (Davis et al., 2023). Additionally, due to cross-reactivity, antigen detection faces considerable challenges in distinguishing between different orthopoxviruses, making protein antigen-based MPXV detection and diagnosis particularly complex (Hughes et al., 2014). The MPXV A29 protein is an important viral envelope protein responsible for mediating the fusion between the virus and the host cell membrane, a mechanism crucial for invasion and infection with the virus (Ye et al., 2023).

The A29 protein is a homolog of the A27 protein in vaccinia virus (VACV), while the A27 protein is required for both Intracellular mature virion (IMV) transport and the process of envelopment that leads to intracellular enveloped virus (IEV) formation (Wang et al., 2023). Research on VACV has shown that the A27 protein binds to glycosaminoglycan (GAG) commonly expressed on the cell surface, which mediates the initial binding between MPXV and the host cells (Ho et al., 2005). Surface plasmon resonance (SPR) technology further demonstrated that MPXV A29 protein can bind to GAG, including heparin and chondroitin sulfate/dermatan sulfate (Shi et al., 2022). The epitope (21-49 aa) on MPXV A29 protein is adjacent to the GAG binding region and is the target of monoclonal antibody (named 69-126-3-7) (Hughes et al., 2014; Wang et al., 2023). Monoclonal antibody mAb 69-126-3-7 is a monoclonal antibody against MPXV prepared by Roumillat et al. (1984). The mAb 69-126-3-7 exhibits a strong reaction with MPXV but does not react with the smallpox virus, cowpox virus, or other smallpox virus isolates, indicating that it can specifically recognize MPXV. Subsequently, scientists tested the cross reactivity of mAb 69-126-3-7 with various orthopoxviruses (Hughes et al., 2014), and the protein that reacted with mAb 69-126-3-7 was identified as MPXV A29 protein, which indicates that monoclonal antibodies targeting A29 protein can be developed to specifically detect MPXV.

In this study, MPXV A29 protein and two other poxvirus proteins highly homologous to A29 protein (CPXV 163 protein homolog and VACV A27 protein homolog) were expressed in *E. coli*, and recombinant A29 protein was used to developed monoclonal antibodies against A29 protein. Using two simulated proteins as reverse screening systems, a pair of monoclonal antibodies specific to the A29 protein were identified through a double antibody sandwich ELISA. Subsequently, CLEIA and colloidal gold immunochromatography assays were developed for detecting A29 protein, providing a foundation for a rapid, cost-effective immunoassay method for the early diagnosis of MPXV.

## 2 Materials and methods

### 2.1 Main materials

*E. coli* competent cells (BL21 Star) and myeloma cell lines (SP2/6) were donated by Professor Xia Ningshao from Xiamen University; A29 protein coding sequence was chemically synthesized by GenScript Biotech Corp.; 6–8 weeks old BALB/c mice were purchased from Hunan SJA Laboratory Animal Co., Ltd. Fetal bovine serum (FBS) and DMEM medium were purchased from TransGen Biotech; Hypoxanthine thymidine (HT), hypoxanthine aminopterin thymidine (HAT), and polyethylene glycol 1500 (PEG-1500) were all purchased from Sigma Aldrich company; GAM-IgG 1-HRP, GAM-IgG 2a-HRP, GAM- IgG 2b-HRP, GAM-IgG 3-HRP, GAM-IgM-HRP, and GAM-IgG-HRP were donated by Professor Xia Ningshao from Xiamen University; streptomycin magnetic beads, alkaline phosphatase, and chemiluminescence substrate solution were all purchased from Hunan Yuanjing Biotechnology Co., Ltd.; other chemicals used are of molecular biology grade or higher, purchased from Sangon Biotech (Shanghai) Co., Ltd.; the PVC substrate, fiberglass film, and absorbent paper were all purchased from Shanghai Jinbiao Biotechnology Co., Ltd.

### 2.2 Ethical approval

The animal operation was approved by the Ethics Committee of Hunan Normal University, with ethics approval number 2024 (D2024032), and was carried out in accordance with the National Institutes of Health Laboratory Animal Care and Use Guidelines (NIH Publications No. 8023, revised in 1978).

### 2.3 Bioinformatics analysis of A29 protein

The A29 protein amino acid sequences from different MPXV subtypes were downloaded from the NCBI website, along with sequences highly homologous to the A29 protein from other poxviruses (including cowpox virus, vaccinia virus, buffalopox virus, camelpox virus, variola virus, etc.). These sequences were analyzed using BioEdit software. The physicochemical properties of the A29 protein were assessed using Protparam analysis software, while antigenic epitopes of the A29 protein were predicted using DNAstar software.

### 2.4 Preparation of recombinant protein

The coding sequence of the A29 protein (Sequence ID: OQ729808.1) was downloaded from the National Center for Biotechnology Information (NCBI) website. A comparative analysis of the amino acid sequences of A29 and other orthologous proteins from poxviruses was performed to identify amino acid differences. Key nucleotide substitutions were introduced to achieve the corresponding amino acid changes. The coding sequences for three proteins (A29 protein, CPXV 163 protein homolog, and VACV A27 protein homolog) were determined, with a 6 × His tag added at the 3′ end for purification purposes. The pTO-T7 vector was used for expression, and a recombinant plasmid was constructed. Codon optimization and chemical synthesis of the recombinant plasmid were carried out by Genescript Biotech Corporation.

The constructed recombinant plasmids were transformed into competent *E. coli* (BL21 Star) cells, and the recombinant strain was induced to express the target protein by adding Isopropyl β-D-thiogalactoside (IPTG) at a final concentration of 1.0 mmol/L. The culture was incubated at 37°C with agitation at 200 rpm for 4 h.

The induced recombinant strain was lysed using an ultrasonic disruptor under ice bath conditions, with 70% power, 3 s of ultrasound followed by a 7-second pause, for a total of 30 min. The supernatant was then subjected to initial purification by ammonium sulfate precipitation, followed by further purification using High Affinity Ni-Charged Resin FF (Lot No. L00666, GenScript Biotech Corporation). The purified sample was subsequently dialyzed against PBS solution. The dialyzed sample was collected and analyzed for purity by SDS-PAGE. The recombinant protein, containing a 6 × His tag, was identified by Western Blot using an anti-6 × His mAb as the primary antibody. Protein concentration was determined using a BCA assay kit.

### 2.5 Preparation of mAb

The recombinant A29 protein was emulsified with an equal volume of Freund’s adjuvant and administered to 6-week-old mice via subcutaneous injection at a dose of 80 μg per mouse. Immunization was performed every two weeks for a total of four injections, with each injection containing 100 μg of recombinant protein per mouse. Three days after the fourth immunization, tail vein blood samples were collected from the mices, and the serum antibody titers were measured using an indirect ELISA.

Mice with the highest serum antibody titers after the final immunization were selected, euthanized using CO2, and their spleens were harvested. The spleen was homogenized in a glass mortar to prepare a single-cell suspension, which was then fused with myeloma cells using PEG-1500 as a fusion reagent. The fused cells were cultured in DMEM containing 20% fetal bovine serum and HAT medium. After cell fusion, hybridoma cells were subcloned using limiting dilution. Following three rounds of selection, a hybridoma cell line producing monoclonal antibodies against the recombinant A29 protein was successfully established.

At 8 weeks of age, female BALB/c mice were intraperitoneally injected with 500 μL of mineral oil. One week later, well-growing hybridoma cells were collected and injected intraperitoneally at a dose of 1 × 106 cells per mouse. After 7–10 days, ascitic fluid was collected. The samples were centrifuged at 5,000 g for 10 minutes at 4°C to remove the precipitate. The supernatant was collected and purified using ammonium sulfate precipitation followed by protein A affinity chromatography. The purified antibodies were identified by SDS-PAGE.

### 2.6 Western blot

The purified recombinant protein was separated by SDS-PAGE and transferred onto a PVDF membrane. After blocking the membrane with 5% defatted milk powder for 2 h and washing, anti-A29 mAb or anti-His mAb (at a concentration of 0.2 μg/mL) were applied as the primary antibody. The secondary antibody, goat anti-mouse horseradish peroxidase (GAM-HRP), was diluted 1:10,000. Chemiluminescent detection was performed using the ECL substrate kit (Lot No. BL520A, Lanjieke Technology Co., Ltd.).

### 2.7 Enzyme-linked immunosorbent assay

#### 2.7.1 Indirect ELISA

The recombinant protein was diluted to 1 μg/mL in carbonate-bicarbonate buffer (15 mmol/L Na2CO3, 35 mmol/L NaHCO3, pH 9.6) and coated onto a 96-well plate overnight. After blocking with 1% BSA solution, the plate was prepared for further use. Anti-A29 monoclonal antibody or hybridoma cell supernatant was diluted to the appropriate concentration and applied as the primary antibody. The plate was incubated at 37°C for 30 min, washed, and then goat anti-mouse horseradish peroxidase (GAM-HRP) secondary antibody was diluted 1:10,000 and added. After a second 30-min incubation at 37°C and washing, TMB substrate was added for color development. The optical density (OD) at 450 nm/630 nm was measured using Microplate Reader. A positive result was defined as an OD_450nm/630nm_ value greater than that of the negative control by more than 2.1 times.

#### 2.7.2 Sandwich ELISA

The process of conjugating monoclonal antibodies with HRP was conducted as outlined in previous studies (Zheng et al., 2023). In the experiment, an ELISA plate was initially coated with the capture antibody (2 μg/mL) and incubated overnight at 4°C. After washing, the plate was blocked with 1% BSA for 2 h. Sandwich antigens (recombinant A29 protein, CPXV 163 protein homolog, and VACV A27 protein homolog) were prepared in 1% BSA at concentrations of 10,000, 1,000, 100, 50, 25, 10, and 0 pg/mL. A 100 μL aliquot of each antigen dilution was added to the wells and incubated for 30 min. Following washing, 100 μL of HRP-conjugated antibodies, diluted 1:5,000, was added and incubated for another 30 min. Color development and readings were performed as described in Indirect ELISA.

### 2.8. Chemiluminescence enzyme immunoassay method

The method for conjugating antibodies with magnetic beads or alkaline phosphatase (AP) follows the procedure outlined in previous studies (Liao et al., 2021).

The preparation of simulated positive samples is carried out according to the following steps: collect MPXV negative serum samples, dilute the A29 protein, CPXV 163 protein homolog and VACV A27 protein homolog with 1% BSA to ten times the required concentration for the experiment, and then add the diluted protein to the negative serum sample in a ratio of 1:9 as a simulated positive sample.

The testing procedure is as follows: add 25 μL simulated serum samples containing A29 protein, CPXV 163 protein homolog, or VACV A27 protein homolog to each incubate tube. Then, add 100 μL 0.04 mg/mL AP-labeled antibody and incubate for 300 sec, followed by three washes. Next, add 50 μL 0.2 mg/mL magnetic bead-conjugated antibody, incubate for another 300 s, and wash three times. Finally, add 200 μL chemiluminescence substrate solution, and measure the relative luminescence value (RLU) of the reaction using a RangeCL-600i chemiluminescence immunoassay analyzer.

### 2.9 Colloidal gold immunoassay

Colloidal gold particles were prepared using the classical citrate reduction method. After conjugating the colloidal gold particles with monoclonal antibodies, the colloidal gold test strip was assembled according to the method described in Figure 7A. Specifically, the nitrocellulose membrane was adhered to the PVC substrate, and the capture antibody was marked at a concentration of 1.0 mg/mL to form the detection line. The GAM-IgG antibody, also at a concentration of 1.0 mg/mL, was used to mark the quality control line. Subsequently, the sample pad, processed bonding pad, and absorbent pad were arranged on the PVC substrate and cut into test strips with a width of 0.3 cm using a slitter for subsequent testing.

The treatment of simulated positive samples is carried out according to the method in Section 2.8. During the experiment, 60 μL of the sample was added to the sample pad, and the strip was incubated for 15 minutes. The detection and quality control lines were then observed.

## 3 Results and analysis

### 3.1 Bioinformatics analysis of monkeypox virus A29 protein

**FIGURE 1:**
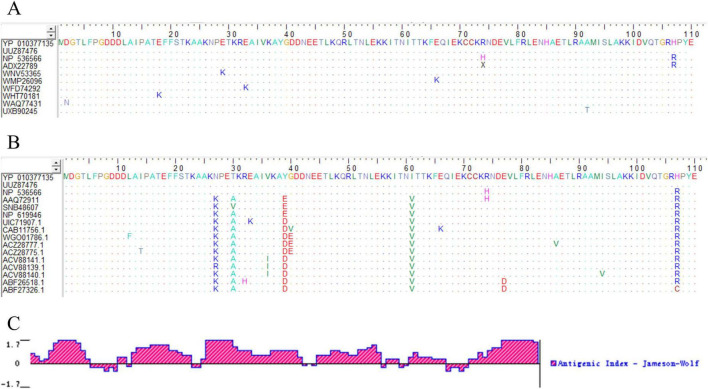
Bioinformatics analysis of A29 protein. **(A)** Homology comparison of amino acid sequences of A29 protein from different MPXV subtypes: The colored letters in the figure represent the amino acid composition of the A29 protein, with different colors indicating different types of amino acids. Dots represent amino acids that are identical to those in the first row of the sequence (the recombinant A29 protein expressed in this study), highlighting the differing amino acids. **(B)** Comparative analysis of the amino acid sequences of MPXV A29 protein and other highly homologous poxvirus proteins: the amino acid sequences in rows 1–3 are from Monkeypox virus, rows 4–6 from Cowpox virus, rows 7–9 from Vaccinia virus, rows 10–11 from Buffalopox virus, rows 12–14 from Camelpox virus, and rows 15–16 from Variola virus. **(C)** Analysis of antigenic epitopes of A29 protein: the X-axis represents the position of the amino acids in the sequence, the Y-axis represents the antigenicity score for each amino acid position, with higher values indicating stronger antigenicity.

Homology analysis of MPXV A29 proteins from different subtypes was performed using BioEdit software, revealing over 95% sequence identity (Figure 1A; Supplementary Figure S1). At the same time, amino acid sequences of other poxviruses (including Cowpox virus, Vaccinia virus, Buffalopox virus, Camelpox virus, Variola virus, etc.) were collected and compared with the amino acid sequence of MPXV A29 protein with BioEdit software. The results indicated that, despite high overall amino acid sequence homology among poxvirus genera, unique amino acids specific to the A29 protein were identified (Figure 1B). This suggests that the A29 protein may contain MPXV-specific epitopes, making it a potential target for MPXV detection. Physicochemical analysis of the MPXV A29 protein revealed a molecular weight of 12.6 kDa. It is a stable, hydrophilic protein with a half-life of over 10 hours in *E. coli*, indicating suitability for prokaryotic expression. Antigen epitope analysis revealed that the MPXV A29 protein contains multiple epitopes along its entire length (Figure 1C), further supporting its potential as an antigen for monoclonal antibody preparation.

### 3.2 Expression and purification of recombinant protein

**FIGURE 2:**
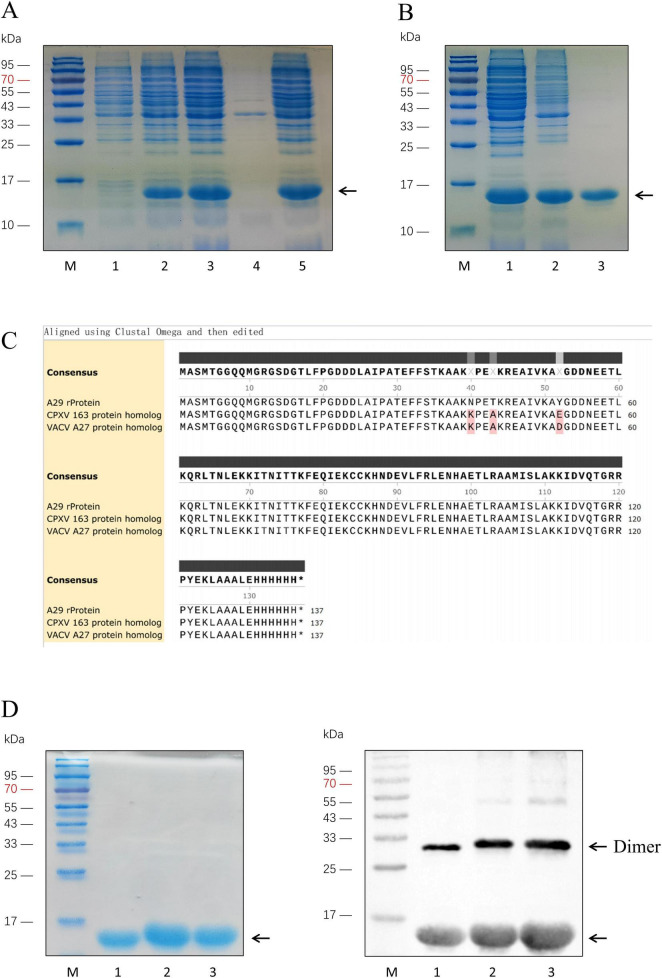
Expression and purification of A29 protein and its homologous proteins. **(A)** Identification of recombinant A29 protein expression: Lane M: Protein relative molecular weight markers; Lane 1: Before induction of A29 protein-expressing *E. coli* strain; Lane 2: After induction of A29 protein-expressing *E. coli* strain; Lane 3: Suspension of A29 protein-expressing *E. coli* strain after sonication; Lane 4: Pellet of A29 protein-expressing *E. coli* strain after sonication; Lane 5: Supernatant of A29 protein-expressing *E. coli* strain after sonication. **(B)** Purification of recombinant A29 protein: Lane M: protein relative molecular weight markers; Lane 1: Protein before ammonium sulfate precipitation; Lane 2: Protein after ammonium sulfate precipitation; Lane 3: Protein after His-tag affinity chromatography purification. **(C)** Comparison of amino acid sequences of the three recombinant proteins: The sequences were derived from the translation of sequencing results of the recombinant plasmids with the T7 promoter from transformed colonies. **(D)** Identification of three purified proteins: Left: Coomassie Brilliant Blue staining; Right: Western Blot identification; Lane M: Protein relative molecular weight markers; Lane 1: Recombinant A29 protein; Lane 2: CPXV 163 protein homolog; Lane 3: VACV A27 protein homolog.

Select the MPXV strain (MPXV/COD/2023/172V, Sequence ID: OQ729808.1) with a well-established research background, and optimize the codons of the A29 protein coding sequence (nt 139871–14203) according to *E. coli* codon preferences. Construct the prokaryotic expression vector pTO-T7-A29, with a 6 × His tag added to the C-terminus of the MPXV A29 protein. Transform the plasmid into *E. coli* (BL21 Star) competent cells and induce expression with IPTG. SDS-PAGE analysis of the induced sample revealed a protein band around 15 kDa, which is slightly larger than A29 protein (12.6 kDa). This increase in molecular weight is due to the 6 × His tag and the linker sequence added between the A29 coding region, consistent with the theoretical molecular weight, indicating successful expression of the recombinant A29 protein. After ultrasonic treatment of the induced samples, SDS-PAGE showed that the recombinant protein was expressed in the supernatant (Figure 2A). The supernatant was collected and subsequently purified using ammonium sulfate precipitation followed by His-tag affinity chromatography. SDS-PAGE analysis confirmed that the recombinant A29 protein was highly pure and suitable for animal immunization (Figure 2B).

Analysis of the amino acid sequences of the A29 protein and its homologs from poxviruses indicated that the differences between A29 and the other homologous proteins are primarily concentrated at the 27th (N-K mutation), 30th (T-A mutation), 39th (Y-E/D mutation), and 61st (I-V mutation) amino acid positions. Given the relatively weak antigenicity of the amino acid at position 61, this residue was left unmodified in the subsequent experiments. Based on the above analysis, we modified several bases in the previously constructed prokaryotic expression vector pTO-T7-A29 (Table 1) to facilitate the expression of the desired protein. In this study, two proteins homologous to A29 protein were selected for the screening of monoclonal antibodies with a reverse screening system: the simulated protein with the 39th amino acid mutated to glutamic acid was named the VACV A27 protein homolog, while the simulated protein with the 39th amino acid mutated to aspartate acid was named the CPXV 163 protein homolog. The coding sequences for the VACV A27 protein homolog and CPXV 163 protein homolog were chemically synthesized and inserted into prokaryotic expression vectors, pTO-T7-VACV A27 protein homolog and pTO-T7-CPXV 163 protein homolog. These vectors were then transformed into *E. coli* (BL21 Star) competent cells, which were cultured overnight. Single colonies were selected and sequenced. We translated the sequencing results into amino acid sequences and compared the sequences of the three proteins (Figure 2C). The results confirmed that the sequences were consistent with the expectations. Colonies matching the expected sequence were selected for recombinant protein expression. SDS-PAGE and Western blot analysis confirmed that the molecular weights of the CPXV 163 protein homolog and VACV A27 protein homolog matched the expected values (Figure 2D). In addition, Western Blot results also indicated that the clear reaction band with a relative molecular weight of 30 kDa and 45 kDa, which is the protein bands resemble dimers and trimers. Previous studies have shown that the smallpox virus proteins, goat pox virus proteins and buffalopox virus proteins homologous to A29 protein are also oligomer (Kumar et al., 2015; Dashprakash et al., 2019). The VACV A27 protein has oligomerization motifs (Strong coil-coil motifs, CCMs) near the carboxyl terminus, which can mediate the formation of protein oligomers. Similarly, it can be inferred that the reason for the appearance of oligomers in A29 protein expression may be related to this.

**TABLE 1:** Differential amino acids between homologous of two other viruses and the MPXV A29 protein as well as their mutated codons and corresponding amino acids.

Amino acid site		27	30	39
CPXV 163 protein homolog	Differential amino acids	N → K	T → A	Y → E
	Corresponding substituted bases	AA**T** → AA**G**	**A**CT → **G**CT	**T**A**T** → **G**A**A**
VACV A27 protein homolog	Differential amino acids	N → K	T → A	Y → D
	Corresponding substituted bases	AA**T** → AA**G**	**A**CT → **G**CT	**T**A**T** → **G**A**C**

### 3.3 Preparation of monoclonal antibodies against recombinant A29 protein

**FIGURE 3:**
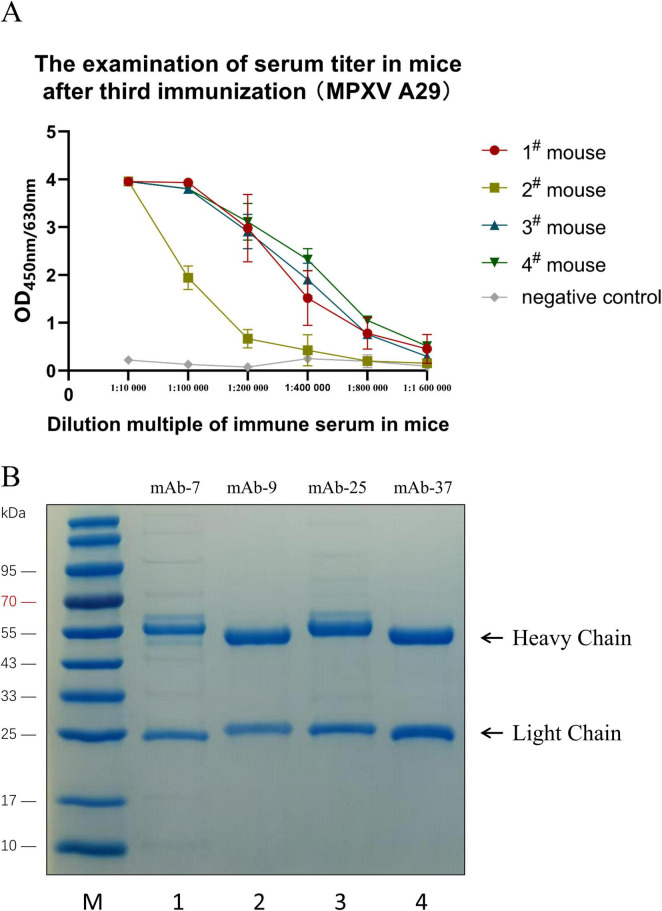
Preparation and purification of monoclonal antibodies. **(A)** Detection of mouse serum titer by indirect ELISA. **(B)** SDS-PAGE analysis of the purified monoclonal antibody: Lane M: Protein molecular weight markers; Lane 1: mAb-7; Lane 2: mAb-9; Lane 3: mAb-25; Lane 4: mAb-37.

Subcutaneous immunization was performed on mice using purified recombinant A29 protein. The initial immunization dose was 100 μg of A29 protein per mouse, and the subsequent three booster immunization doses were all 60 μg of A29 protein per mouse, with 14 days interval between each immunization. Three days after the third booster immunization, blood samples were collected from the tail vein of the mice, and serum antibody titters were measured using the indirect ELISA. The results are shown in Figure 3A: after three booster immunizations, the serum titers of mice with No. 1, 3, and 4 reached 1:1 600,000 while the serum titer of the mouse with No. 2 reached 1:400,000, which indicated that the prepared recombinant protein has excellent immunogenicity.

The spleen from immunized mice was fused with SP2/6 myeloma cells. After three rounds of subcloning and screening, four hybridoma cell lines secreting monoclonal antibodies against the recombinant A29 protein were successfully obtained: mAb-7, mAb-9, mAb-25, and mAb-37. Subsequently, these four hybridoma cells were injected into the peritoneal cavity of sensitized mice with paraffin oil to prepare ascites. The ascitic fluid antibody was purified using ammonium sulfate precipitation and protein A affinity chromatography. The purified antibodies were analyzed using SDS-PAGE, and the results showed that all the antibodies exhibited two bands: the band at 50 kDa represents the heavy chain of the antibody, while the band at 25 kDa represents the light chain. No other non-specific bands were observed, indicating successful antibody purification (Figure 3B).

### 3.4 Property of monoclonal antibodies against A29 protein

**FIGURE 4:**
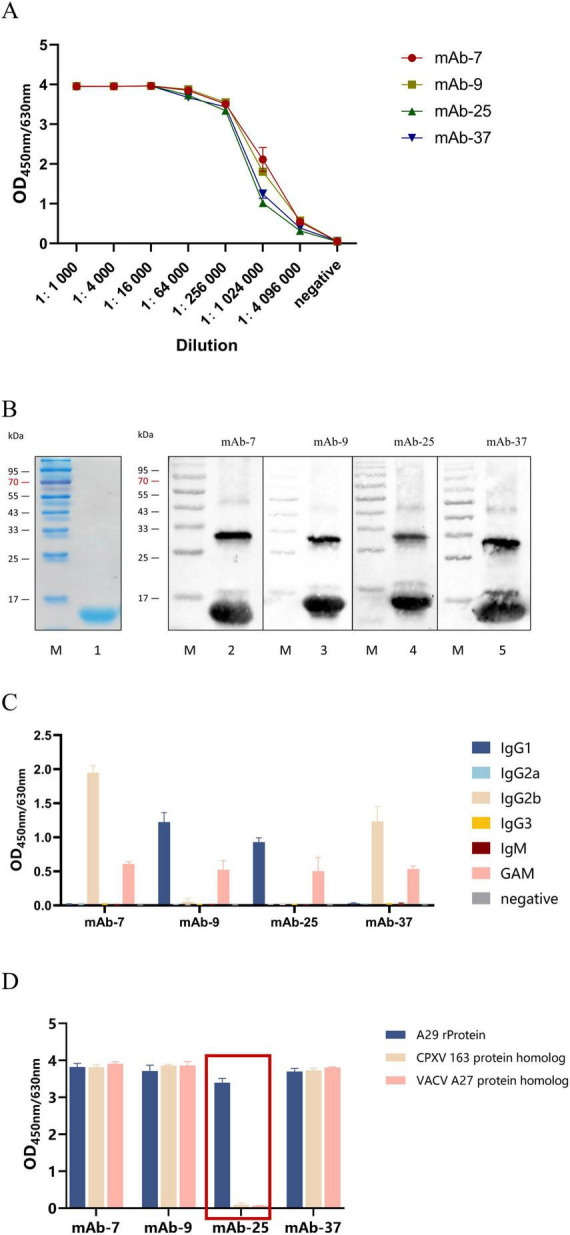
Characterization of monoclonal antibodies. **(A)** Determination of monoclonal antibody titer by indirect ELISA. **(B)** Western Blot analysis of the reactivity of monoclonal antibodies with the protein: Lane M: Protein molecular weight markers; Lane 1: Coomassie Brilliant Blue staining to identify A29 protein; Lanes 2–5: Western Blot identification of A29 protein using mAb-7, mAb-9, mAb-25, and mAb-37 as primary antibodies, respectively. **(C)** Antibody isotyping. **(D)** Specificity of the antibody determined by indirect ELISA.

Indirect ELISA was used to detect antibody titters. Four monoclonal antibodies were diluted at 1,000, 4,000, 16,000, 256,000, 1,024,000, and 4,096,000 times, respectively, and used as primary antibodies. The results showed that the titers of antibodies 7, 9, 25, and 37 could all reach 1:4,096,000 or even higher, indicating that the screened monoclonal antibodies had a good immunoreactivity (Figure 4A). Four monoclonal antibodies were used as primary antibodies for Western blotting, and the results showed that all four monoclonal antibodies could effectively recognize the recombinant A29 protein (Figure 4B). The subtypes of mouse antibodies mainly include Ig G1, Ig G2a, Ig G2b, Ig G3, and Ig M. Indirect ELISA was used to determine antibody subtypes, the five types of mouse HRP labeled antibodies were used as secondary antibodies and HRP-labeled GAM was used as positive controls. The results showed that mAb-7 and mAb-37 were IgG 2b types, and mAb-9 and mAb-25 were IgG G1 types (Figure 4C). Further, indirect ELISA was used to detect the specificity of four pairs of antibodies. The results indicated that monoclonal antibody mAb-25 only recognized the recombinant A29 protein, and it was a specific monoclonal antibody for recombinant A29 protein. The other three monoclonal antibodies were able to recognize three proteins and had no specificity for recombinant A29 protein (Figure 4D).

### 3.5 Selection of the optimal paired antibody

**FIGURE 5:**
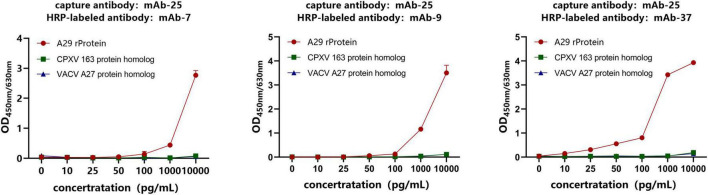
Screening of antibody pairs by sandwich ELISA. The specific antibody mAb-25 was used as the capture antibody, while the non-specific antibodies mAb-7, mAb-9, and mAb-37 were used as HRP-labeled antibodies to establish a sandwich ELISA.

As mentioned above, four monoclonal antibodies against the recombinant A29 protein were obtained. Among them, mAb-25 is a specific antibody against A29, while the other three are non-specific. To establish an immune detection method specific to the recombinant A29 protein, mAb-25 was used as the capture antibody, while the remaining three non-specific antibodies (mAb-7, mAb-9, and mAb-37) were used as HRP-conjugated detection antibodies in a double antibody sandwich ELISA. The results demonstrated that mAb-25 successfully paired with mAb-7, mAb-9, and mAb-37. Notably, the mAb-25/mAb-37-HRP antibody pair showed a higher OD_450nm/630nm_ value at low antigen concentrations (10–1,000 pg/mL) (Figure 5). Therefore, the mAb-25/mAb-37-HRP pair was selected for further evaluation.

### 3.6 Establishment and evaluation of chemiluminescence enzyme immunoassay method

**FIGURE 6:**
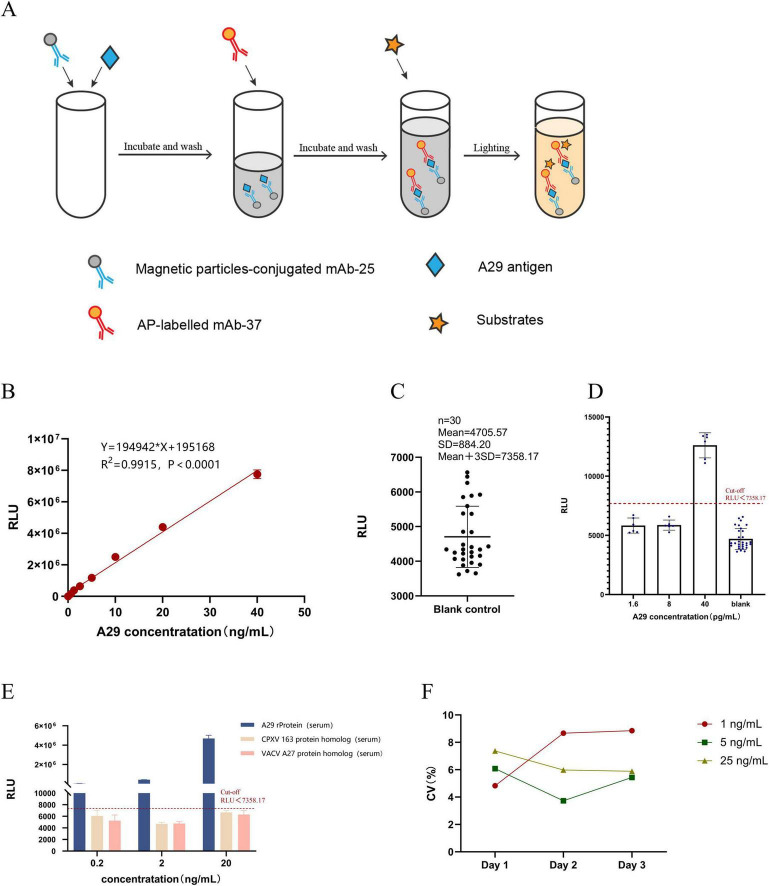
Establishment and evaluation of chemiluminescent enzyme immunoassay. **(A)** Principle of Chemiluminescence Enzyme Immunoassay Method. **(B)** Construction of the standard curve: Chemiluminescent enzyme immunoassay was used to measure different concentrations of simulated positive samples, and a linear regression analysis was performed between RLU readings and sample concentration. **(C)** Determination of the cut-off value: Thirty blank samples were measured, and the cut-off value was set as the mean + 3SD. **(D)** Detection limit determination: The concentration of recombinant A29 protein in simulated positive serum samples was adjusted to different values, and the test was repeated six times. **(E)** Specificity testing: Simulated positive serum samples (recombinant A29 protein, VACV A27 protein homolog, and CPXV 163 protein homolog) were prepared at three different concentrations (20, 2, and 0.2 ng/mL), with each sample measured in triplicate to determine the RLU value. **(F)** Stability testing: Simulated positive serum samples (recombinant A29 protein) were prepared at low, medium, and high concentrations. Each sample was measured over three consecutive days, with three replicates per day, to calculate the coefficient of variation.

The principle of the chemiluminescence method is shown in Figure 6A. A series of simulated samples with known concentrations (0.625, 1.25, 2.5, 5, 10, 20, and 40 ng/mL) were prepared. The simulated clinical samples were then analyzed using the established chemiluminescence enzyme immunoassay, with each sample tested in triplicate. Finally, GraphPad Prism software was use for linear regression fitting. The results, as shown in Figure 6B, indicate that the established detection method has a good linear relationship (*R*^2^ = 0.9915) within the concentration range of 0.625–40 ng/mL.

Measure the RLU values of 30 negative serum samples using the established CLEIA and take the mean plus 3 times the standard deviation (SD) as the cut-off value. Simulated positive serum samples with concentrations of 1.6, 8, and 40 pg/mL were prepared, followed by measurement of their RLU values using the established CLEIA. Repeat the test 6 times for each concentration of sample. The results showed that the cut-off value of the CLEIA was 7358.17, with a detection limit of 40 pg/mL (Figures 6C,D).

Simulated positive serum samples containing recombinant A29 protein, CPXV 163 protein homolog, and VACV A27 protein homolog were prepared at concentrations of 20, 2, and 0.2 ng/mL, followed by detection using the established CLEIA. Each sample was repeated three times. The results showed that, despite the CPXV 163 protein homolog, VACV A27 protein homolog, and recombinant A29 protein differing by only three amino acids, the established CLEIA could effectively distinguish between the three proteins (Figure 6E). This indicates that the method exhibits high specificity for recombinant A29 protein in serum samples.

Simulated positive serum samples containing recombinant A29 protein were prepared at low, medium, and high concentrations of 1, 5, and 25 ng/mL, respectively. The samples were detected using the established CLEIA with a frequency of three times per day for three consecutive days, and their RLU values were recorded. The coefficient of variation (CV) of the RLU values obtained from daily testing at each concentration was then calculated. The results showed that the CV values for each concentration were below 10% per day (Figure 6F), indicating that the CLEIA established in this study exhibits high precision and good repeatability.

### 3.7 Establishment and evaluation of colloidal gold immunoassay

**FIGURE 7:**
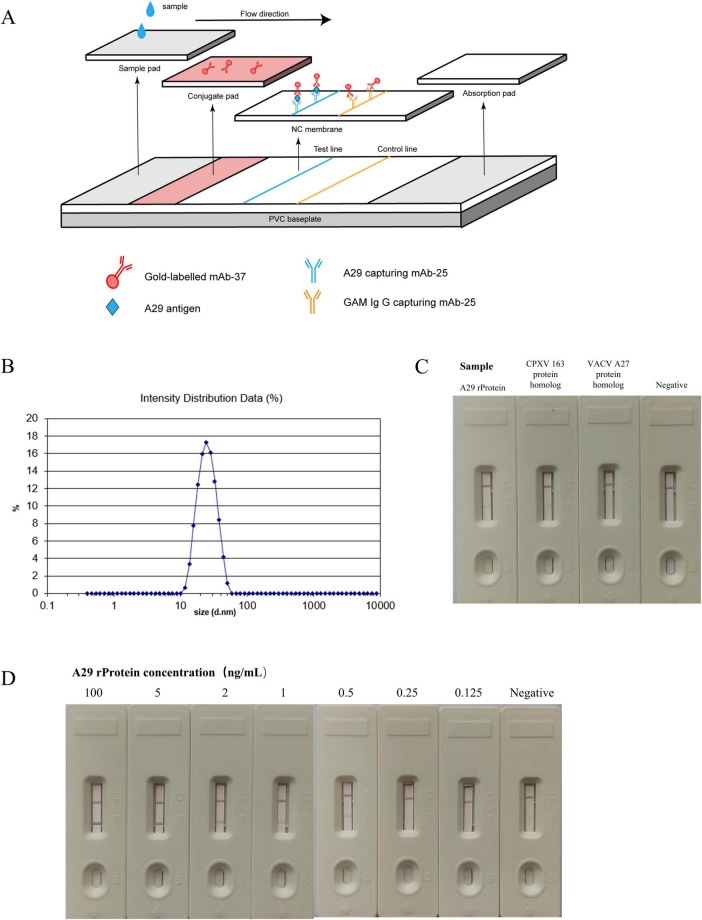
Establishment and evaluation of colloidal gold immunochromatographic assay. **(A)** Schematic diagram of the colloidal gold immunoassay. **(B)** Measurement of the particle size of gold nanoparticles. **(C)** Specificity test of colloidal gold test strips: the reactivity of colloidal gold test strips with recombinant A29 protein, VACV A27 protein homolog, and CPXV 163 protein homolog was measured. **(D)** Sensitivity test of colloidal gold test strips: the reactivity of test strips with samples containing different concentrations of recombinant A29 protein was evaluated.

The assembly method of the colloidal gold test strip in this section is shown in Figure 7A. Firstly, dynamic light scattering (DLS) analysis was used to measure the particle size of the customized colloidal gold, which was found to be approximately 20 nm (Figure 7B). The colloidal gold exhibited a rose-red color, making it suitable for antibody labeling. After labeling the antibody, it can be assembled into an immunogold label test strip. The effectiveness test results of the gold-labeled test strip demonstrated its ability to specifically recognize the recombinant A29 protein, with no immune reactivity to the CPXV 163 protein homolog or VACV A27 protein homolog (Figure 7C). This indicates that the test strip exhibits excellent specificity. Simultaneously, the sensitivity of the test strip was evaluated, and its detection limit was found to be 0.25 ng/mL, indicating that the test strip demonstrates good sensitivity to the simulated serum sample (Figure 7D).

## 4 Discussion

MPXV is a large DNA virus with a genomic structure that shares a high degree of homology with other poxviruses. Therefore, the rational selection of detection targets is crucial for subsequent virus infection detection. This study aims to establish a highly specific immunodiagnostic method for MPXV. Given the high homology between MPXV and other poxviruses, it is essential to ensure that the selected antigen targets are specific to MPXV. Researcher Roumillat developed the monoclonal antibody mAb 69-126-3-7, which could specifically targets MPXV by recognizing the A29 protein as its antigenic epitope (Roumillat et al., 1984; Hughes et al., 2014). In subsequent studies, Ye et al. (2023) analyzed the amino acid sequence recognized by this antibody and conjugated the peptide to KLH, which was then used to immunize mice for the production of specific monoclonal antibodies. They ultimately succeeded in obtaining a monoclonal antibody that specifically recognizes the MPXV. However, due to the low immunogenicity of the peptide, the monoclonal antibodies produced by Liya and his colleagues had insufficient sensitivity, which may result in some missed detections. These research suggest that the A29 protein is a reliable and specific antigen target.

This study first performed a comprehensive analysis of the amino acid sequence of the A29 protein, revealing its excellent water solubility and confirming its classification as a soluble protein. Moreover, the relatively long half-life of A29 protein in *E. coli* makes it an efficient, rapid, and cost-effective method for its expression. Considering these factors, the study chose to use a prokaryotic expression system to express the recombinant A29 protein, which was subsequently used for immunization of mice. After three booster immunizations, the serum titters of several mice reached over 1:1,600,000, indicating that the recombinant A29 protein expressed in the prokaryotic system possessed strong immunogenicity. In comparison to peptides, the recombinant protein demonstrated a more potent ability to induce a robust immune response in mice. Subsequently, splenocytes from the immunized mice were fused with cultured myeloma cells. After three rounds of subcloning and screening, four hybridoma cell lines secreting antibodies against the recombinant A29 protein were successfully obtained. The reactivity of these four antibodies with the A29 protein was also assessed using indirect ELISA and Western Blot. The results demonstrated that all four antibodies exhibited strong immunoreactivity with the A29 protein, laying a solid foundation for the development of efficient immunodiagnostic methods.

To screen for monoclonal antibodies specifically recognizing the A29 protein, researchers typically use homologous proteins from other poxviruses, such as the 163 protein from cowpox virus and the A27 protein from vaccinia virus, as negative selection systems (Ye et al., 2023; Liang et al., 2024). Due to the minimal amino acid differences and similar physicochemical properties of these proteins, the same expression and purification methods as those used for A29, such as prokaryotic expression followed by nickel affinity chromatography, can be employed. This study provides a more comprehensive analysis: our research employed a bioinformatics analysis to compare A29 protein with homologous proteins from viruses such as CPXV and VACV, aiming to screen monoclonal antibodies that specifically recognize A29 protein without cross reaction with related poxvirus proteins. Through sequence alignment, we identified key amino acid differences among the three viral proteins, particularly at site 27 (N-K mutation), 30 (T-A mutation), 39 (Y-E/D mutation), and 61 (I-V mutation). Consequently, the homologous genes from cowpox and vaccinia viruses were synthesized for further investigation. Given that the antigenicity of the amino acid I at position 61 in the A29 protein is relatively low, we chose to express the homologous proteins from cowpox and vaccinia viruses with the same I at position 61 as in the MPXV. Thus, two A29 homologous proteins were identified as negative selection systems: the one with a mutation at position 39 to glutamic acid (E) was named CPXV 163 protein homolog, and the one with a mutation at position 39 to aspartic acid (D) was named VACV A27 protein homolog. After successfully expressing and purifying recombinant A29 protein, CPXV 163 protein homolog and VACV A27 protein homolog, we evaluated the reactivity of four monoclonal antibodies with the three proteins using indirect ELISA. The results demonstrated that mAb-25 specifically recognized the recombinant A29 protein without cross-reacting with homologous proteins from the other two poxviruses. As a result, mAb-25 was selected as the capture antibody, while the other three antibodies were used as enzyme-labeled antibodies in pairwise assays. Fortunately, all three antibody pairs demonstrated successful compatibility. To identify the most effective antibody pair for detection, this study employed a sandwich ELISA to assess antigen detection at different concentrations. Ultimately, the mAb-25/mAb-37-HRP antibody pair exhibited superior detection performance at lower antibody concentrations. Consequently, we selected the mAb-25/mAb-37-HRP antibody pair as the foundation for developing an immunodiagnostic assay.

Chemiluminescent enzyme immunoassay (CLEIA) is a high-throughput, rapid, and highly sensitive immunoassay technique. It is widely applied in clinical diagnostics, public health surveillance, as well as the detection of specific molecules, such as proteins, nucleic acids, hormones, and pollutants, in environmental samples. This technique relies on the chemiluminescent signal generated by the chemical reaction between enzyme-labeled antibodies and substrates, with the signal intensity quantified using a chemiluminescence analyzer (Dowlatshahi et al., 2021). In this study, we used mAb-25 conjugated to magnetic beads as the capture antibody and mAb-37 conjugated to alkaline phosphatase as the enzyme-labeled antibody to establish the CLEIA. Due to biosafety concerns and the difficulty in obtaining MPXV-positive serum, we employed simulated positive serum for validation, adding a known concentration of recombinant A29 protein to negative serum to evaluate the interference resistance of the established CLEIA in complex serum samples. In the preliminary experiments, we used a 1% BSA dilution buffer to prepare antigen samples and generate a standard curve. However, due to interference from serum components, the standard curve did not align with the values from the recombinant protein in the simulated serum samples. To mitigate the impact of serum contaminants on the assay, we modified the approach by diluting recombinant protein in negative serum to known concentrations, establishing a new standard curve. The results showed that the new standard curve demonstrated a strong linear relationship (*R*^2^ = 0.9915) within the concentration range of 0.625–40 ng/mL, which was thus defined as the linear range of the CLEIA. Based on this, the CLEIA was further evaluated and showed good detection performance with a limit of detection of 40 pg/mL. Recombinant A29 protein was diluted to three concentrations (1, 5, 25 ng/mL), and testing was performed three times daily over three consecutive days. The coefficient of variation of the RLU values for each concentration was less than 10%, indicating that the CLEIA developed in this study exhibits good stability and reproducibility. Furthermore, when three antigens (recombinant A29 protein, CPXV 163 protein homolog, and VACV A27 protein homolog) were tested at three concentration gradients (20, 2, 0.2 ng/mL), only the recombinant A29 protein gave positive results. The two non-specific proteins tested negative, further confirming the high specificity of the CLEIA.

The colloidal gold immunochromatographic strip offers a rapid, cost-effective, and highly specific method for detection (Dong et al., 2022). The primary material, colloidal gold particles, can be prepared using a variety of methods, with particle sizes ranging from 5 to 150 nm (Horisberger, 1981; Squarzoni, 2023). In this study, colloidal gold nanoparticles (approximately 20 nm in diameter) were synthesized using the classic citrate reduction method. After optimizing the reaction conditions for the gold nanoparticle labeling, the detection antibody was conjugated with the colloidal gold nanoparticles, and the final gold-labeled test strip was assembled. This strip demonstrated specific recognition of the recombinant A29 protein, without cross-reacting with two other homologous mimetic proteins, indicating excellent immunoreactivity specificity. Further sensitivity testing revealed that the detection limit could reach 0.25 ng/mL.

In this study, the CLEIA method was developed using a monoclonal antibody targeting the A29 protein as the detection probe. Compared to the biosensor developed by Can et al. (2024), which uses heparin sulfate protein as the detection probe and has a detection limit of 2.08 ng/mL, our CLEIA method demonstrates a lower detection limit, indicating that monoclonal antibodies offer higher sensitivity as detection probes for antigen recognition. When compared with the TRFIA (Time-Resolved Fluorescence Immunoassay) method developed by Yan et al. (2024), which also uses monoclonal antibodies as the detection probe, the CLEIA method in this study achieves a comparable detection limit. Additionally, it not only ensures high sensitivity but also exhibits the ability to specifically recognize the A29 protein of MPXV. Building upon this, a colloidal gold immunochromatographic method was established using mAb-25 and mAb-37 antibodies. This method combines high sensitivity and specificity with advantages such as rapid detection, no need for complex instruments, and visual result interpretation. This highlights that the antibodies selected in this study demonstrate strong performance across multiple detection systems, and the established method shows promising sensitivity and specificity. In the future, improvements can be made to the system and reaction conditions to further enhance the detection of the A29 protein of MPXV.

Due to biosafety concerns, this study did not use actual MPXV-positive serum to evaluate the developed immunoassay method, and therefore, its effectiveness in samples from MPXV-infected individuals remains uncertain. Conditions permitting, we plan to further validate the method using positive serum samples.

In conclusion, this study successfully developed four monoclonal antibodies targeting the A29 protein, with one monoclonal antibody, mAb-25, identified as a MPXV-specific monoclonal antibody. It was successfully paired with other monoclonal antibodies. The selected monoclonal antibodies exhibited excellent detection performance on both the CLEIA and colloidal gold immunochromatographic platforms, demonstrating potential for clinical MPXV detection.

## Data Availability

The raw data supporting the conclusions of this article will be made available by the authors, without undue reservation.

## Footnotes

1 World Health Organization (2024a).

2 World Health Organization (2024b).
