# Supplementary material for: Preparation and application evaluation of monoclonal antibodies against Monkeypox virus A29 protein
Source: Front Microbiol. 2025 Jan 31;16:1547021. doi: 10.3389/fmicb.2025.1547021 (PMC11825512; doi:10.3389/fmicb.2025.1547021)
Supplement: Supplementary file 1 [file Data_Sheet_1.docx]

Supplementary Material

# Supplementary Figures and Tables

## Supplementary Figures


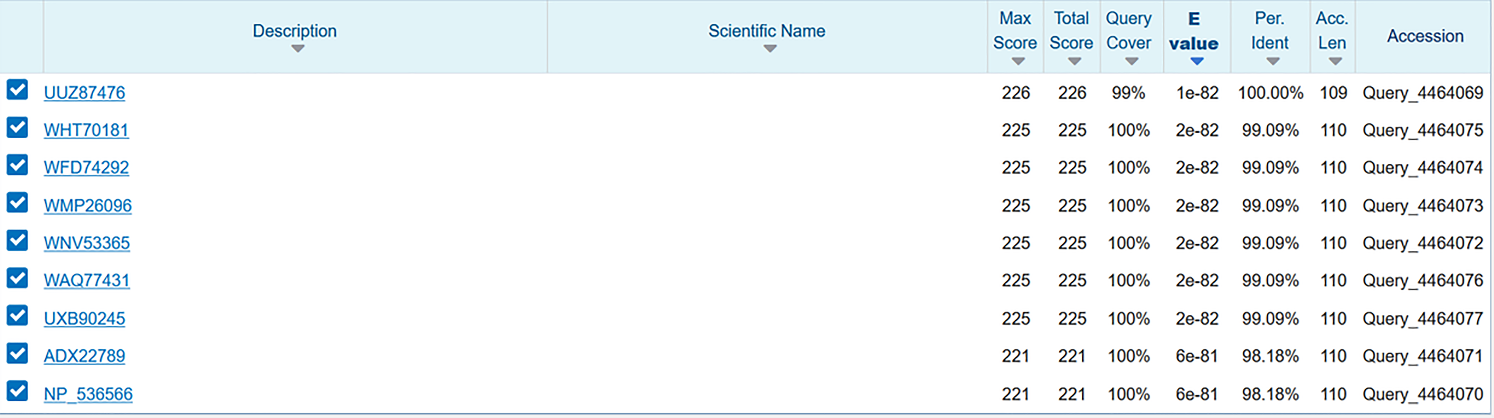


**Supplementary Figure 1.** Sequence identity: The percentage identity (Per. Ident) of other amino acid sequences was calculated using YP_010377135 as the reference.
